# Posttransplant diabetes mellitus and long-term outcomes after kidney transplantation in a steroid avoidance regimen: a cohort study

**DOI:** 10.1186/s12882-025-04419-2

**Published:** 2025-09-26

**Authors:** Claire Gardiner, David Keane, Eva Ho, Virginie Lenfant, Ozlem Tankisi, Michelle Hiu Ching Yam, Sunil Daga

**Affiliations:** 1 Nutrition and Dietetic Group, Rehabilitation and Health Professions, School of Health, Leeds Beckett University, Leeds, LS1 3HE UK; 2 University of Galway, Galway, Ireland; 3 Dietetic Department, Nottingham University Hospitals NHS Trust, Nottingham, UK; 4 Dietetic Department, Bradford Teaching Hospitals Foundation Trust, Bradford, UK; 5 Dietetic Department, Barts Health NHS Trust, London, UK; 6 Dietetic Department, Heartlands Hospital – University Hospitals Birmingham NHS Foundation Trust, Birmingham, UK; 7 Renal Medicine, Leeds Teaching Hospitals NHS Trust, Leeds, UK

**Keywords:** PTDM, Transplant, Obesity, Steroid avoidance, Patient survival, Death censored graft survival

## Abstract

**Background:**

Posttransplant diabetes mellitus (PTDM) is associated with reduced patient survival and death-censored graft survival and has been linked to steroid use and obesity. Steroid avoidance regimens have been associated with a reduction in PTDM without impacting patient or graft survival followed up for 5 years following kidney transplantation; however, acute rejection remains a concern. The primary objective of this study was to assess death-censored graft and patient survival outcomes and report on PTDM onset in patients receiving steroid-avoidance immunosuppressant regimens over 11 years.

**Methods:**

This was a retrospective cohort study from a single center in the UK that included first kidney transplants between 2010 and 2021. Logistic regression models and Cox proportional hazards models were used to investigate associations between survival and PTDM. A P value < 0.05 was considered to indicate statistical significance.

**Results:**

There was no difference in patient or graft survival among those with PTDM, preexisting diabetes or no diabetes. 16% (*n* = 55) developed PTDM over a median follow-up of 7.1 years (range: 0.9–13.8 years). After adjusting for confounding factors, the odds of PTDM diagnosis were associated with increasing BMI (odds ratio (OR): 1.01; 95% CI: 1.03–1.18), and white ethnicity was associated with reduced odds of PTDM (OR: 0.45; 95% CI: 0.23–0.90).

**Conclusions:**

Our findings support lower PTDM rates and safe longer-term outcomes following a steroid-free regimen. Timely weight management interventions before transplantation, particularly in high-risk groups, may reduce PTDM in this population.

**Clinical trial number:**

Not applicable.

**Supplementary Information:**

The online version contains supplementary material available at 10.1186/s12882-025-04419-2.

## Background

Solid organ transplantation is the predominant mode of treatment in patients with end-stage kidney disease (ESKD), far exceeding all other renal replacement therapy (RRT) options, where 56% of adults living with ESKD were transplanted by the end of 2020 in the UK [1] and increasing in proportion to the others every year [2, 3]. The prognosis of kidney transplants, however, can vary, with first-time transplant graft survival ranging from 86 to 92% at 5 years, depending on whether a deceased or living donor is received [2]. A yearly median decline in the eGFR of -0.8 ml/min/1.73 m^2^ is typically observed after the first year after transplantation [1]. The decline in kidney transplant function can be due to many factors related to recipient health for example, such as hypertension [4], but, more notably, metabolic complications posttransplantation, including posttransplant diabetes mellitus (PTDM) [5, 6].

PTDM, defined as new-onset and persistent hyperglycaemia 3 months posttransplant in transplant recipients and present for more than 6 months [7–9], has characteristics and pathophysiological mechanisms similar to those of type 2 diabetes, e.g., insulin resistance or a decline in the production of insulin [10]. However, treatment differs with insulin typically being advocated as a first-line treatment for hyperglycaemia posttransplantation to rest the pancreas and avoid the development of PTDM [11]. The reported prevalence of PTDM in recipients of solid organ transplants varies between 9% and 40% [12], which may be reflective of the diagnostic criteria used, the era of immunosuppression and the changing demographics of kidney transplant recipients. One in three individuals on the kidney transplant waiting list are from ethnic minority communities, with some transplant centers having more than 50% of their list as per the NHS Blood and Transplant report from 2023 to 24 [2]. Modifiable and nonmodifiable determinants of PTDM have been well documented [13–15], as well as their impact on long-term health outcomes. Nonetheless, obesity and immunosuppressants have also been associated with long-term outcomes such as patient survival and death-censored graft loss [16, 17]. There are conflicting data on the long-term consequences of PTDM, but this has been attributed to heterogeneous cohorts and methodological differences [18].

Considerable changes have evolved in immunosuppressant regimens over the years to reduce the risk of these regimens but preserve kidney transplantation. Over 40% of regimens in the U.S. are steroid avoidance or minimisation [19] and attempt to develop the withdrawal of calcineurin inhibitors (CNIs) [20]. Steroid-free or avoidance studies from the UK have demonstrated safety over relatively short follow-up periods [21, 22] and are actively pursued in centres with a relatively large proportion of patients from ethnic minority communities [23]. The concerns of acute rejection, death-censored graft loss and patient survival in kidney transplant recipients receiving steroid avoidance or steroid withdrawal regimens have been dispelled to a certain degree [13, 21, 24, 25]; however, confidence remains low. While the Cochrane review supports these concerns, there is an acknowledgement that information in this cohort beyond 5 years is lacking [26].

With research indicating that a BMI > 30 kg/m^2^ increases the risk of developing PTDM irrespective of any subsequent weight gain [27, 28], lifestyle interventions involving weight management posttransplant have been studied [29–33]. However, there has been limited research on the impact of either preventing or delaying PTDM, where only the “Comparing glycaemic benefits of active versus passive lifestyle intervention in kidney allograft recipients” (CAVIAR) study was able to show that an active lifestyle intervention halved PTDM diagnosis (*p* = 0.123) and resulted in weight loss (*p* = 0.023); however, this was a small cohort sample, and the study time was short [32]. Pretransplant management of obesity is recommended by both national [1] and international [34] guidelines because of associated poor surgical outcomes as well as poor long term outcomes including increased risk of PTDM [35]. In contrast, however, findings from a cohort study (*n* = 464) revealed that living with obesity pretransplant did not impact physical health-related quality of life posttransplant. It is important to note though that this research did not explore quality of life with weight gain posttransplant [36].

The dietetic workforce in the UK [37] is currently unable to meet the recommendations of regular dietetic intervention in this cohort [38]. Therefore, more research is needed to explore the most effective and efficient way of addressing this issue, particularly at what time point, in relation to transplantation, an intervention would be of impact.

As a result, the primary objective of this observational study was to describe the incidence of PTDM and identify risk factors of PTDM in recipients of first kidney transplantation following steroid avoidance regimen from the outset in a real-life setting over an 11-year period.

Secondary objective of this study was to assess death-censored graft and patient survival outcomes in patients receiving steroid-avoidance immunosuppressant regimens over 11 years in recipients of first kidney transplantation stratifying for diabetes status.

## Methods

### Study design

This was a retrospective cohort study in which data were routinely collected from a single center in the UK. Information governance approval was obtained through a local regulatory process (Data Access Committee). University Ethics [39] was obtained to use data for undergraduate and master-level studentships. Informed consent was not obtained as the previously collected data were fully anonymised and used for secondary analysis only. We excluded data for individuals who opted out via the NHS National Data Opt-Out service. The study is reported following the recommendations of the Strengthening the Reporting of Observational Studies in Epidemiology guidance [40] (see appendix 1 for the checklist).

### Data extraction

We included data from individuals under the care of the Leeds Renal Transplant Service who underwent first-time kidney transplantation between 01/01/2010 and 31/12/2021. We extracted data from the renal clinical information system, VitalData (VitalPulse, Chelmsford, UK), including relevant demographic and clinical data, laboratory variables (such as random blood sugar (RBS), HbA1c), medications and outcome information. We excluded individuals who had previously received a kidney transplant, who were no longer under the care of the Leeds Renal Service 5 years posttransplant, where the transplanted kidney failed before 5 years or where they were less than 18 years of age at the date of transplantation.

### Definition of variables

#### Longitudinal data

We collected longitudinal data at timepoints defined as 3 and 6 months (t=-3 and t =-6) before the date of transplant; at transplantation (t = 0); and at 3, 6, 9, 12, 24, 36, 48 and 60 months posttransplant (t = 3–60). Data were included at t = 0 if they were recorded between one month pretransplant and one day posttransplant. For timepoints between t=-6 and t = 9, we included data from within +/-1 month of the time point, and for those between t = 12 and t = 60, we included data from within +/-3 months.

#### Pretransplant diabetes (PreDM)

We included patients with PreDM on the basis of a recorded comorbidity in the clinical information system, documented diabetic medication preceding the date of transplant or a recorded random blood glucose ≥ 11.1 mmol/l prior to transplantation.

#### PTDM

We defined PTDM as having a first recorded prescription of oral hyperglycaemic agents or insulin continuing at or beyond t = 3 months or after or having RBG ≥ 11.1 mmol/l from t = 3 months that remained elevated without any medication.

#### Transient hyperglycaemia

Where RBG ≥ 11.1mmo/l was noted at 0–3 months, no further hyperglycaemic readings were recorded beyond 3 months and/or if any ongoing treatment was required after 3 months, this was categorised as transient hyperglycaemia.

#### Steroid-avoidance immunosuppression

Individuals whose prednisolone prescription was not recorded at time points t = 0 and t = 3 months.

#### Uncontrolled hypertension

Individuals with a median systolic blood pressure between months t=-6 and t = 0 greater than 130 mmHg.

### Posttransplant clinical care

Our unit uses a steroid avoidance regimen such that individuals posttransplant receive only IV methylprednisolone as part of induction therapy along with almetuzumab at the time of transplantation surgery [22]. Steroids are included only if there are additional risks, such as preexisting autoimmune disease or increased immunological risk of rejection. The local trust guidelines use the following treatment for posttransplant hyperglycaemia and PTDM: RBG ≥ 11.1 mmol/l, where the patient is asymptomatic, leads to the commencement of an oral antihyperglycemic agent, such as biguanide, sulphonurea or Dipeptidyl peptidase-4 (DDP-4) inhibitors. Where the patient is symptomatic or has RBG > 15 mmol/l or RBG > 25 mmol/l 3 months after transplantation, insulin therapy is initiated. The dietetic services offered to the individuals varied over time.

### Data analysis

We summarised the baseline variables, stratified by diabetes status, using means (standard deviations) and proportions as appropriate. To investigate the associations between clinical and demographic variables and PTDM onset, we constructed logistic regression models, where the development of PTDM during follow-up was the outcome. We used Cox proportional hazards models to consider whether PTDM onset was associated with patient survival and with death-censored graft survival. Statistical analyses were performed via R version 4.2.3 [41], and any p value < 0.05 was considered statistically significant.

## Results

### Study cohort

**Fig. 1:**
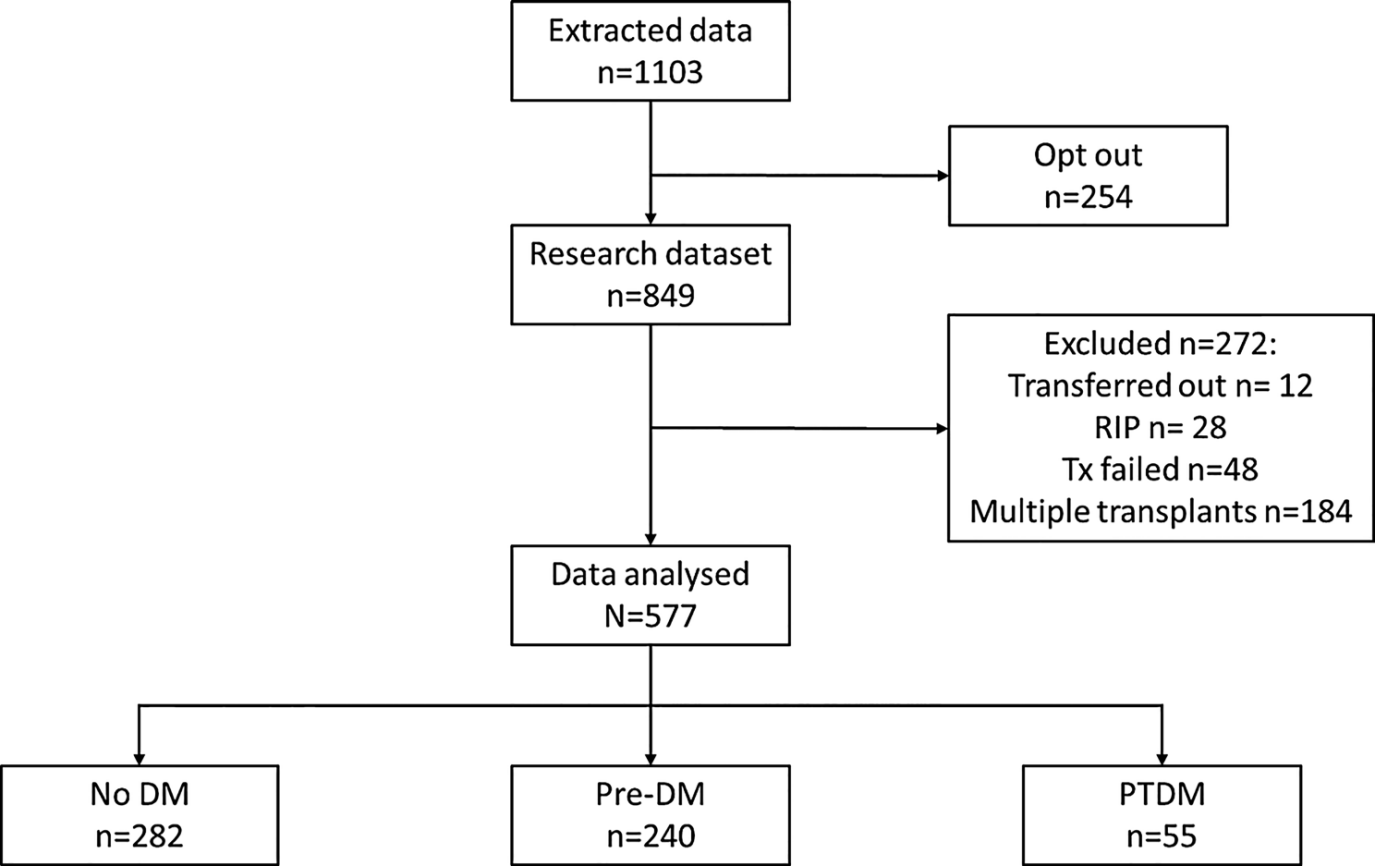
Study flow diagram

The extracted dataset included records of 1053 individuals, 254 associated with patients who had opted out of their data being used for research and 272 excluded as per the exclusion criteria, leaving 577 individuals with a first-time kidney transplant meeting the criteria for the study (Fig. 1). Data were complete (0% missing data) for demographic data, including age, sex, ethnicity and transplant records are maintained up to date and audited for completion. Missingness was low for weight and blood pressure data (< 10%) at all time points up the 48 and 60 month timepoints where missingness rose to around 30%, likely due to loss to follow up. Missingness was higher for blood glucose data ranging from about 20% missing in early follow up to around 40% around the end of cohort follow up.

The baseline (t = 0) characteristics are shown in Table 1. Compared to those with diabetes status pre transplant and no diabetes, individuals with PTDM tended to be male, less likely to be from white ethnic groups, have uncontrolled hypertension, have a higher baseline BMI and be more likely to have received steroids as part of their immunosuppressant regimen (Table 1). A total of 81% of patients were maintained on a steroid free immunosuppression regime. Compared to those who were prescribed steroids, this group were less likely to have uncontrolled hypertension (61% compared to 66%), had higher BMI (27.1 compared to 26.6), more likely to be male (63% compared to 60%) and more likely to be younger age (51 years compared to 52).

**Table 1:** Characteristics of the total cohort and by DM status. Data are presented as percentages or means (SDs) for normally distributed variables

	All	PTDM	PreDM	No-DM
n	577	55	240	282
Age (years)	51.0 (14)	54.2 (13)	52.3 (13)	49.3 (16)
Ethnicity White	77%	65%	73%	82%
Sex (male)	63%	71%	63%	60%
Hypertension	71%	71%	71%	71%
BMI (kg/m^2^)	27.0 (4.6)	28.6 (4.8)	27.3 (4.5)	26.5 (4.7)
Steroid-avoidance immunosuppression	81%	73%	82%	82%
12 month % weight gain	3.1 (7.0)	4.1 (6.6)	2.5 (6.4)	3.4 (7.5)

### Incidence of PTDM

According to our definition, 55 of 577 (10%) individuals in the overall cohort whose allograft lasted 5 years or more, developed PTDM during a median follow-up of 7.1 years (range 0.9–13.8 years). The incidence among individuals with no prior history of DM (PTDM) is 16%. Among those 55 patients, 37 (67%) were categorised on the basis of RBG values or diabetic medication use within the first year posttransplant, 7 (13%) were categorised on the basis of data recorded between one and two years posttransplant, and 11 (20%) were categorised after the end of the second year. Prescriptions for diabetic medications were documented for 46 of the 55 patients (85%).

### Risk factors for PTDM

Increasing baseline BMI and non-white ethnicity were positively associated with the odds of PTDM development in unadjusted analyses, and these associations were maintained in adjusted analyses (see Table 2 for covariates), with steroid avoidance immunosuppression associated with reduced odds of PTDM. No statistically significant associations with PTDM were found for age, sex, hypertension or weight gain (Table 2).

**Table 2:** Odds ratios for the risk of developing PTDM

	Unadjusted OR (95% CI)	Adjusted OR (95% CI)
Age (years)	1.02 (1.00-1.04)	1.02 (0.99–1.04)
Ethnicity (white* vs. non white)	0.54* (0.30–0.99)	0.45* (0.23–0.90)
Sex (Male)	1.51 (0.84–2.85)	1.37 (0.72–2.73)
Hypertension	1.46 (0.77–2.96)	1.16 (0.58–2.50)
BMI (kg/m^2^) **	1.09* (1.02–1.16)	1.10* (1.03–1.18)
Steroid avoidance immunosuppression	0.60 (0.33–1.16)	0.48* (0.24–0.97)
12 month % weight gain	1.02 (0.98–1.07)	1.03 (0.99–1.08)

*White ethnicity used as the reference group for odds ratio calculation

** BMI refers to baseline (T0) measurement

### Associations between PTDM and outcomes

**Fig. 2:**
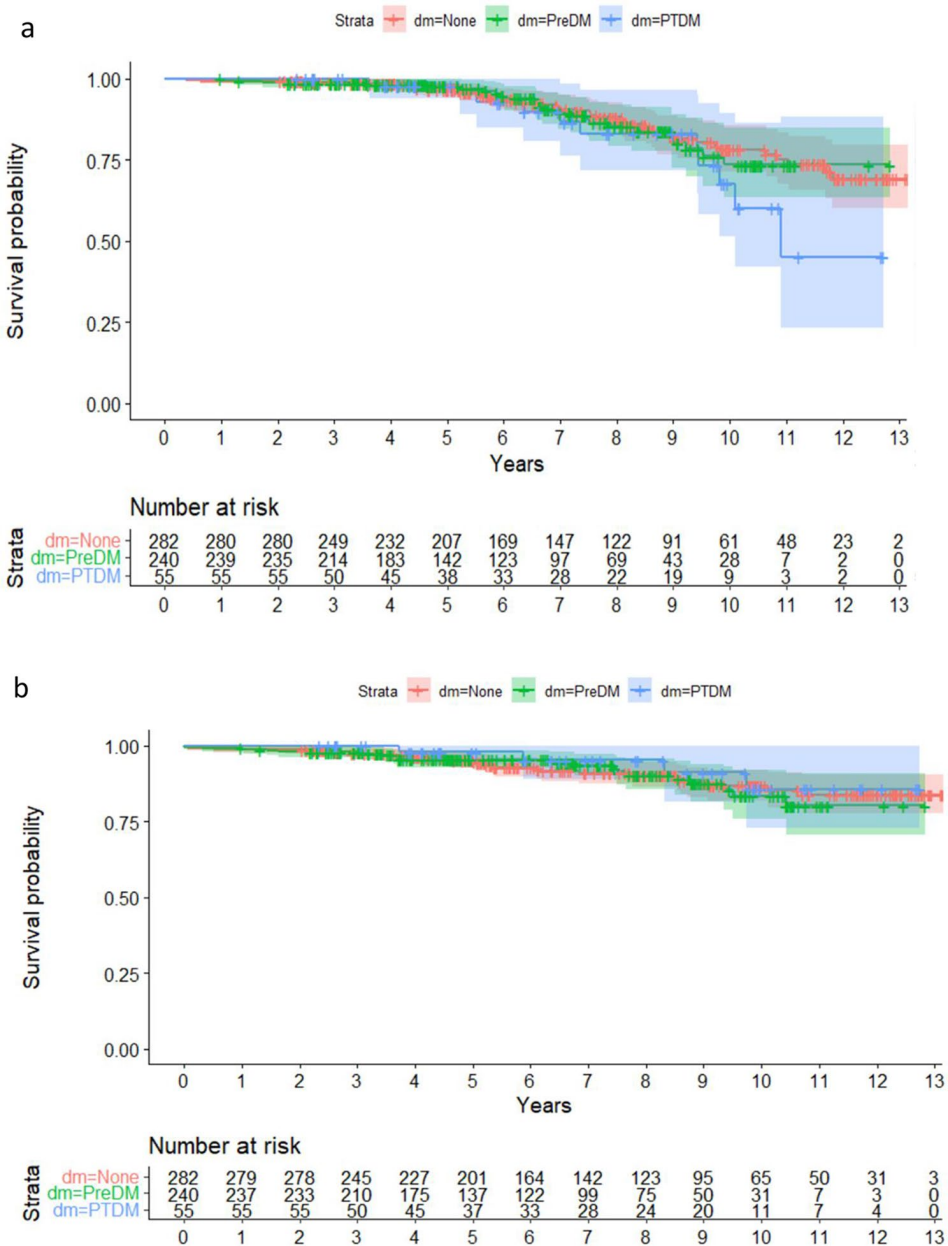
**a**: Kaplan‒Meier curves for patient survival by diabetes status. **b**: Survival curves for death-censored graft survival by diabetes status

We compared outcomes for those individuals with PTDM status, those who were living with diabetes before their transplant and those with no evidence of diabetes. The ten-year survival rate across all individuals was 76%, with rates of 78% for individuals without diabetes, 73% for individuals living with diabetes preceding the transplant and 68% for those with PTDM. When considering graft survival, 80% of the cohort had functioning grafts at ten years, including 79% of those living with PTDM and 74% of those living with diabetes preceding transplantation (Table 3). Kaplan-Meier analysis revealed no statistically significant differences in these outcomes based on diabetes status, although the wide confidence intervals were consistent with relatively small subgroups (Fig. 2a and b). We did not find any evidence that PTDM was a risk factor for either graft failure or patient survival, as the median follow-up durations were 6.2 and 6.7 years, respectively (Table 4). Younger age and male sex may be associated with graft failure, whereas older age may be associated with worse patient survival. Steroid avoidance may not be associated with poor patient or graft survival.

**Table 3:** One-, three-, five- and ten-year mortality and graft failure rates by diabetes status

		No-diabetes	Pre-DM	PTDM	All
Patient survival rate	1-year	100% (98–100)	100% (100–100)	100% (100–100)	100% (99–100)
	3-year	99% (98–100)	99% (97-0.00)	100% (100–100)	99% (98–100)
	5-year	97% (95–99)	98% (96–100)	98% (94–100)	97% (96–99)
	10-year	78% (71–85)	73% (64–85)	68% (51–89)	76% (70–81)
Death censored graft survival rate	1-year	99% (98–100)	100% (100–100)	100% (100–100)	100% (98–100)
	3-year	99% (98–100)	99% (98–100)	100% (100–100)	99% (98–100)
	5-year	97% (94–99)	98% (96–100)	97% (92–100)	97% (96–99)
	10-year	82% (75–90)	74% (64–87)	79% (63–100)	80% (74–86)

**Table 4:** Cox proportional hazards model for the risk of graft failure/mortality

	Graft failure		Mortality	
	Unadjusted HR	Adjusted HR	Unadjusted HR	Adjusted HR
DM status (vs. no diabetes) - Pre-DM - PTDM	1.06 (0.59–1.91) 0.74 (0.26–2.13)	1.75 (0.87–3.48) 0.97 (0.28–3.39)	1.10 (0.66–1.83) 1.58 (0.81–3.09)	1.06 (0.59–1.90) 1.63 (0.79–3.35)
Age (years)	0.97* (0.96-1.00)	0.97* (0.95–0.99)	1.10* (1.08–1.13)	1.11* (1.08–1.14)
Ethnicity* (white vs. non white)	0.75 (0.40–1.39)	0.97 (0.46–2.04)	0.89 (0.53–1.52)	0.67 (0.34–1.30)
Sex (Male)	0.65 (0.38–1.13)	0.50* (0.27–0.95)	1.44 (0.89–2.35)	1.39 (0.81–2.39)
Hypertension	0.71 (0.40–1.28)	1.04 (0.52–2.10)	1.00 (0.60–1.68)	1.10 (0.60-2.00)
BMI**	1.00 (0.94–1.06)	1.04 (0.97–1.11)	1.02 (0.97–1.07)	1.02 (0.97–1.09)
Steroid avoidance immunosuppression	0.85 (0.42–1.69)	0.79 (0.36–1.76)	0.68 (0.40–1.17)	0.57 (0.31–1.06)
12 month % weight gain	1.02 (0.97–106)	1.01 (0.97–1.06)	0.97 (0.94–1.01)	0.98 (0.94–1.03)

*White ethnicity used as the reference group for hazards ratio calculations

** BMI at baseline (T0) used in this model

Data were censored for death. The median follow-up time for the mortality model was 6.7 years, and that for graft failure was 6.2 years.

## Discussion

PTDM is an additional morbidity many patients can experience following kidney transplantation [18]. Steroid avoidance is one of the proposed ways to reduce PTDM, but concerns about longer-term outcomes remain unanswered. In this paper, we present data with much longer follow-up periods than those of any previous publication looking at patients receiving steroid avoidance immunosuppressant regimens, which revealed that 16% of the cohort developed PTDM over eleven years posttransplant, with 67% being diagnosed within the first year following transplantation. White ethnicity, lower BMI and a steroid avoidance immunosuppression regimen may be associated with lower odds of PTDM onset. We did not observe any associations between PTDM and patient survival or death-related graft failure in this study.

The incidence of PTDM observed in this study compares well with that reported by Ekberg et al. [25], where 12.4% of the cohort following the steroid avoidance regimen was diagnosed with PTDM. Our findings are similar, as 11.8% (*n* = 40) of the individuals who were receiving steroid avoidance immunosuppressant regimens were diagnosed with PTDM despite a much longer follow-up period.

Our findings confirm that high BMI and non-white ethnicity remain independent significant risk factors for PTDM onset despite steroid avoidance. Compared with other studies [13–15], this study did not find any significant difference in age or sex, although a greater proportion of PTDM cases in our cohort were male, and the mean age of the PTDM cohort was 51 years. Importantly, however, our demographics align with the national picture in this cohort [1]. This lack of significance, however, may have been due to the small sample size in contrast to other studies. A recent study suggested that older age, apart from a higher baseline BMI, was significantly associated with PTDM over five years of follow-up; however, there was no breakdown of ethnic groups, suggesting that this may be a confounding factor [42].

The mean BMI of the group of individuals with PTDM was 28.6 kg/m^2,^ with a mean weight gain of 4.1 kg over the first year, which aligns well with findings from other studies [27, 43]. Forte et al. [28] suggested a lower prevalence of PTDM in a group that had more than 5% weight gain in the first year; however, their study predominantly included individuals of white ethnicity (89%), and the group with weight gain had a lower baseline BMI. Our data also revealed that posttransplant weight gain is not a major risk factor for PTDM; however, baseline BMI is. A higher baseline BMI in our cohort was associated with an increased risk of PTDM, likely through the same mechanisms of insulin resistance as in the general population; however, PTDM is precipitated following CNI exposure [5, 6]. The results of this study raise the issue of when it is best to intervene with weight management advice. The rationale for weight management pretransplant tends to align with transplant listing and surgical risks rather than with PTDM [44]. However, based on our cohort with a mean BMI of 28.6 kg/m^2^, many patients were not considered for weight loss interventions before transplantation for two reasons. First, the cut-off for most units in the UK for transplant listing is up to 35 kg/m^2^, unless it is considered for a pancreas and kidney transplant where the BMI is reduced to 30 kg/m^2^. Second, a BMI of 28 kg/m^2^ in the dialysis population is considered to be protective in comparison to the general population. This is referred to as the obesity paradox [45, 46].

As a result, research has focused on posttransplant weight management, monitoring HbA1c and serum glucose as secondary outcomes of intervention [30–33]. The CAVIAR study demonstrated that active lifestyle intervention resulted in significant improvement in weight and halved PTDM rates but did not reach statistical significance. The study had good representation of non-white ethnicities (32.3%) but was not powered to study PTDM outcomes [32]. The evidence for posttransplant dietary intervention to prevent PTDM is of lower quality, with one systematic review suggesting that a Mediterranean and vegetarian diet is associated with lower PTDM risk [47]. There is a real need for prospective intervention studies to inform evidence-based interventions for the prevention of not only PTDM but also other metabolic complications associated with weight following kidney transplantation.

It is well understood in the literature that modifiable risk factors include immunosuppressant therapy, where both corticosteroids and CNI are known to have a diabetogenic effect in transplant recipients. In our study, patients who were treated with steroid-avoidant immunosuppression were less likely to be diagnosed with PTDM than were those treated with steroids after adjusting for confounding factors. These results are consistent with both a cohort study [48] and a randomised control trial [25] demonstrating the protective effect of steroid avoidance immunosuppressant regimens on PTDM onset. Ekberg et al. [25] reported a higher incidence of PTDM in the steroid maintenance group, where they further reported that 40% of the patients in the steroid avoidance group resolved their PTDM diagnosis within two years posttransplant than did 28.5% in the steroid maintenance group. Although this is not an area we have explored within our data, it does support our theory. Our cohort was managed by Alemtuzumab induction and had mycophenolate and steroids added on the basis of immunological risk. A highly sensitive patient managed with a steroid-free regimen and tacrolimus monotherapy can increase the risk of PTDM associated with the treatment of episodes of rejection [49]; thus, tailoring the immunosuppressant regimen to competing risks is needed.

This study reviewed patient outcomes, observing death censored graft survival and mortality data for up to 11 years, adjusting for modifiable and nonmodifiable risk factors for PTDM onset. When outcomes were compared with those of patients living with diabetes before transplant and patients without diabetes status, there was no significant difference at the median follow-up of 6.2 years and 6.7 years. Our findings compare well with those of Ekberg et al. [25], who reported a follow-up of 2 years in a steroid avoidance cohort, and Malik et al. [5], who reported a median follow-up of 6 years, even with their cohort receiving maintenance doses of prednisolone. A meta-analysis by Lin et al. [16] suggested that there is a 35% increased risk of graft loss in PTDM patients (7 studies; *n* = 6231; *p* = 0.0002). However, the authors acknowledge caution with these findings, as while they consider PTDM to negatively impact graft survival, the mechanisms of death-censored graft loss are unclear and, in fact, may increase the risk of death with a functioning graft.

On further review of our findings, although we did not find any statistically significant differences in survival rates, it is pertinent to note that this is based on a relatively small sample size [7]. It is well documented that PTDM has a greater metabolic burden; therefore, an impact on transplant outcomes is plausible [50]. For patients of a younger age, the findings imply a better patient survival outcome, whereas older age or being female was associated with a lower chance of death-censored graft failure in patients who were receiving steroid avoidance immunosuppression. This finding is in line with reported outcomes in England [51].

There are limitations to this study. First, the data collected were from a single center, and only retrospective data were collected, so there was a limitation on what information was available for analysis. While our definition does not align fully with the American Diabetic Association criteria [52] for the diagnosis of PTDM, our criteria for PTDM diagnosis in this cohort were considered, taking them into account as well as considering the local trust policy. The timing of data collection was paramount, as 3 months is a crucial starting point for any posttransplant blood glucose readings in line with PTDM diagnosis. It is recognised that any observed hyperglycaemia within the first three months of transplant may be due to a stress response to surgery [53]. We also observed random blood glucose levels greater than or equal to 11.1 mmol/l, which is reflective of the guidelines. Where we are unable to report symptoms experienced by the patient in conjunction with hyperglycaemia, we noted the prescription of medication typically associated with the management of diabetes. The presence of persistent hyperglycaemia where medication was not prescribed was also acknowledged as a PTDM diagnosis.

Second, there was no information on the dosage of tacrolimus or monitoring of blood tacrolimus levels. Although 40 patients who were receiving a steroid avoidance regimen were diagnosed with PTDM, it was difficult to know what other factors may have contributed beyond the nonmodifiable risk factors. There has been limited exploration of other risk factors for PTDM beyond demographics, where knowing the type of graft (cadaver or living donor) may have added more context. It is important to note that we were unable to include acute rejection episodes or steroid treatment for rejection. As this was a retrospective cohort study, there were limitations to data collection. Treatment was also based on clinical decisions, which may have been influenced by a number of factors which could confound the results, and therefore a possibility of indication bias needs to be considered.

Third, 24% of the patients had opt-out for research, which is 5 times greater than what is observed in England [54]; thus, this may have affected our conclusions, and this is, unfortunately, the nature of observational studies.

There were several strengths in this study where the data presented represented outcomes over 11 years posttransplant (the longest period reported in the literature) and focused on steroid avoidance immunosuppression therapy, which, to the best of the authors’ knowledge, limited research has been published in this area.

In conclusion, this research improves the understanding and evidence supporting the efficacy of the use of a steroid avoidance regimen in kidney transplant recipients, where the risk of death, censored graft and survival outcomes are not increased in the first 11 years posttransplant compared with the literature focused on steroid-inclusive regimens. However, the risk of PTDM onset is still evident, particularly in relation to high BMI, male sex and non-white ethnicity; therefore, PTDM onset should be considered when planning to focus on the management of weight and lifestyle before and after transplantation and the judicious use of steroids in immunosuppression regimens.

## Supplementary Information

Below is the link to the electronic supplementary material.

Supplementary Material 1

## Data Availability

The data that support the findings of this study are available from Dr. Sunil Daga, but restrictions apply to the availability of these data, which were used under licence for the current study and are not publicly available. Data are, however, available from the authors upon reasonable request and with permission of Leeds Teaching Hospitals Trust the Data Analysis Committee.
