# Supplementary material for: Posttransplant diabetes mellitus and long-term outcomes after kidney transplantation in a steroid avoidance regimen: a cohort study
Source: BMC Nephrol. 2025 Sep 26;26:520. doi: 10.1186/s12882-025-04419-2 (PMC12465307; doi:10.1186/s12882-025-04419-2)
Supplement: Supplementary file 1 — Supplementary Material 1 [file 12882_2025_4419_MOESM1_ESM.docx]

APPENDIX 1: STROBE checklist

|  | **Item No** | **Recommendation** | **Page no.** |
| --- | --- | --- | --- |
| **Title and abstract** | 1 | (*a*) Indicate the study’s design with a commonly used term in the title or the abstract | **1** |
|  |  | (*b*) Provide in the abstract an informative and balanced summary of what was done and what was found | **1** |
| **Introduction** | | |  |
| Background/rationale | 2 | Explain the scientific background and rationale for the investigation being reported | **3-5** |
| Objectives | 3 | State specific objectives, including any prespecified hypotheses | **5-6** |
| **Methods** | | |  |
| Study design | 4 | Present key elements of study design early in the paper | **6** |
| Setting | 5 | Describe the setting, locations, and relevant dates, including periods of recruitment, exposure, follow-up, and data collection | **6-8** |
| Participants | 6 | (*a*) *Cohort study*—Give the eligibility criteria, and the sources and methods of selection of participants. Describe methods of follow-up | **6-8** |
|  |  | (*b*) *Cohort study*—For matched studies, give matching criteria and number of exposed and unexposed |  |
| Variables | 7 | Clearly define all outcomes, exposures, predictors, potential confounders, and effect modifiers. Give diagnostic criteria, if applicable | **7-8** |
| Data sources/ measurement | 8* | For each variable of interest, give sources of data and details of methods of assessment (measurement). Describe comparability of assessment methods if there is more than one group | **7-8** |
| Bias | 9 | Describe any efforts to address potential sources of bias | **6-8** |
| Study size | 10 | Explain how the study size was arrived at | **6-8** |
| Quantitative variables | 11 | Explain how quantitative variables were handled in the analyses. If applicable, describe which groupings were chosen and why | **8** |
| Statistical methods | 12 | (*a*) Describe all statistical methods, including those used to control for confounding | **8** |
|  |  | (*b*) Describe any methods used to examine subgroups and interactions | **8** |
|  |  | (*c*) Explain how missing data were addressed | **n/a** |
|  |  | (*d*) *Cohort study*—If applicable, explain how loss to follow-up was addressed | **n/a** |
|  |  | (*e*) Describe any sensitivity analyses | **n/a** |

| **Results** | | |
| --- | --- | --- |
| Participants | 13* | (a) Report numbers of individuals at each stage of study—eg numbers potentially eligible, examined for eligibility, confirmed eligible, included in the study, completing follow-up, and analysed **9-14** |
|  |  | (b) Give reasons for non-participation at each stage **9** |
|  |  | (c) Consider use of a flow diagram **9** |
| Descriptive data | 14* | (a) Give characteristics of study participants (eg demographic, clinical, social) and information on exposures and potential confounders **9-10** |
|  |  | (b) Indicate number of participants with missing data for each variable of interest **n/a** |
|  |  | (c) *Cohort study*—Summarise follow-up time (eg, average and total amount) **9-14** |
| Outcome data | 15* | *Cohort study*—Report numbers of outcome events or summary measures over time |
| Main results | 16 | (*a*) Give unadjusted estimates and, if applicable, confounder-adjusted estimates and their precision (eg, 95% confidence interval). Make clear which confounders were adjusted for and why they were included **9-14** |
|  |  | (*b*) Report category boundaries when continuous variables were categorized **9-14** |
|  |  | (*c*) If relevant, consider translating estimates of relative risk into absolute risk for a meaningful time period |
| Other analyses | 17 | Report other analyses done—eg analyses of subgroups and interactions, and sensitivity analyses **9-14** |
| **Discussion** | | |
| Key results | 18 | Summarise key results with reference to study objectives **15** |
| Limitations | 19 | Discuss limitations of the study, taking into account sources of potential bias or imprecision. Discuss both direction and magnitude of any potential bias **19-20** |
| Interpretation | 20 | Give a cautious overall interpretation of results considering objectives, limitations, multiplicity of analyses, results from similar studies, and other relevant evidence **16-19** |
| Generalisability | 21 | Discuss the generalisability (external validity) of the study results **15** |
| **Other information** | | |
| Funding | 22 | Give the source of funding and the role of the funders for the present study and, if applicable, for the original study on which the present article is based **n/a** |
